# Learning styles and preferences for live and distance education: an example of a specialisation course in epidemiology

**DOI:** 10.1186/1472-6920-13-93

**Published:** 2013-07-02

**Authors:** Rolf HH Groenwold, Mirjam J Knol

**Affiliations:** 1 Julius Center for Health Sciences and Primary Care, University Medical Center Utrecht, PO Box 85500, 3508, GA, Utrecht, The Netherlands; 2 RIVM Center for Infectious Disease Control, Bilthoven, The Netherlands

**Keywords:** Distance Education, Epidemiology, Learning Styles

## Abstract

**Background:**

Distance learning through the internet is increasingly popular in higher education. However, it is unknown how participants in epidemiology courses value live vs. distance education.

**Methods:**

All participants of a 5-day specialisation course in epidemiology were asked to keep a diary on the number of hours they spent on course activities (both live and distance education). Attendance was not compulsory during the course and participants were therefore also asked for the reasons to attend live education (lectures and practicals). In addition, the relation between participants’ learning styles (Index of Learning Styles) and their participation in live and distance education was studied.

**Results:**

All 54 (100%) participants in the course completed the questionnaire on attendance and 46 (85%) completed the questionnaire on learning styles. The number of hours attending live education was negatively correlated with the number of hours going studying distance learning materials (Pearson correlation −0.5; p < 0.001). The most important reasons to attend live education was to stay focused during lectures (50%), and to ask questions during practicals (50%). A lack of time was the most important reason *not* to attend lectures (52%) or practicals (61%). Learning styles were not association with the number of hours spent on live or distance education.

**Conclusion:**

Distance learning may play an important role in epidemiology courses, since it allows participants to study whenever and wherever they prefer, which provides the opportunity to combine courses with clinical duties. An important requirement for distance learning education appears to be the possibility to ask questions and to interact with instructors.

## Background

Distance learning through the internet is increasingly popular in higher education [1,2]. Important reasons for this are the possibility to increase the number of participants that can simultaneously be enrolled in a course and possibly each participant can follow the course at a convenient time. With advances in technology, the number of modes of distance education will probably increase over the coming years. Which mode of education is appropriate, seems to depend on contents, context and participants [1].

Also in the field of epidemiology, different modes of distance education have been introduced, particularly to allow participants to combine courses in epidemiology with other activities, for example clinical duties. However, it is not well known how this transition from live to distance education (i.e., education without face-to-face interaction between participants and instructor) is appreciated by participants of epidemiology courses. In addition, instructors may be ignorant of the potential of distance education. We set out to assess which mode of education (live vs. distance) is preferred by participants in an epidemiology course and what are predictors of potential preferences. Here we describe our experiences with the introduction of distance learning components in a 5-day specialisation course in epidemiology on confounding and effect modification and how this was valued by the course participants. In addition, we studied the relation between participants’ learning styles and their preference for live and distance learning.

## Methods

### Research setting

The research presented here was performed within the annual course ‘Advanced topics in causal research: confounding and effect modification’. This is a course within the MSc program Epidemiology at Utrecht University. It is a compulsory course for MSc Epidemiology students as well as for postgraduate MSc students (mostly PhD students) in Epidemiology. In the curriculum the course is scheduled at the end of a series of theoretical courses, just before the students start their research projects. In addition to the aforementioned groups of participants, the course is attended by clinicians with a research interest.

The course focuses on two issues in epidemiology that are essential to causal research: confounding and effect modification. For a detailed explanation of these topics we refer to textbooks on epidemiology [3]. During the course, approximately half of the time was devoted to the topic of confounding and the other half to effect modification. There were two teachers in this course: one was an expert in the field of confounding, the other an expert in the field of effect modification.

The course is a five-day course. The first four days have a similar set-up: each morning lectures are scheduled in which theory is discussed, while in the afternoon computer practicals are scheduled in which statistical software is used to clarify the theory discussed during the morning session. On the fifth day of the course a group assignment is scheduled. Each group is assigned an empirical dataset and an accompanying research question. The group members have to draft an analysis plan, analyse the data accordingly, and give a short presentation of the results of their study. At the end of the course, the participants have to pass a written exam.

Since 2008, the course has been given annually according to the format described above. In 2012 the outline of the course was similar to the years before, but in addition course materials were developed to allow participants to follow the course through distance learning. In 2011, all lectures were recorded on video. The slides and the timeline of the lectures were added to the recordings, which allowed participants to go easily through the lecture or play specific parts multiple times. In addition, for all computer practicals a video manual was recorded, which includes a video instruction on using the statistical software, how to perform the exercises, where to find the results, and how to interpret these results. All materials were available through the E-learning environment on a website. Students could access the website using a personal code. Aside from the study materials, there was also room to post messages on the E-learning environment. These messages could be read by all participants as well as the instructors. This allowed participants to ask questions or to respond to questions by others. Participants could also send messages directly to the instructors, either through the website or email. During the 2012 course, attendance to the live education was not compulsory and distance learning material were made available in parallel with the live sessions. Hence, participants could choose to attend the live education, to use the learning materials provided through the internet, or both.

### Data collection and analysis

All participants of the course that was given in March 2012 were asked to keep a diary on the number of hours they spent on course activities. They were also asked to write down the reasons for attending or not attending the live education (lectures or practicals). In addition, they were asked to fill in a questionnaire on learning styles. We used the Index of Learning Styles, which was developed by Felder and Soloman [4]. The Index of Learning Styles makes a distinction between four domains of learning (active-reflective, sensing-intuitive, visual-verbal, and sequential-global). The questionnaire consists of 44 questions, 11 for each domain. For each participant the answers to the questionnaire were categorized to indicate which was the predominant learning style in each of the four domains. All questionnaires were answered voluntarily and anonymized before they were collected. Therefore, we were unable to link the results of the exam to e.g. attendance of live or distance education or learning styles. The study was approved by the NVMO Ethical Review Board (NERB dossier number 261).

## Results

All 54 participants in the course filled in the questionnaire on attendance of live and distance education. The majority (34/54 = 63%) of the participants were female, the mean age was 28.4 years (sd 4.9), and 41 (76%) participants followed the course as part of the postgraduate MSc Epidemiology program. Prior to the course, the mean duration participants were active on the internet per day was 3.6 hours (sd 2.2) and 27 (50%) of them had never been enrolled in a course that included some form of distance education.

The mean time participants spent on this course was 33.0 hours (sd 7.6). Twenty (37%) participants attended all lectures (i.e., 4 lectures) and all practicals (i.e., 3 practicals) during the course. Only 5 (9%) participants did not attend any lecture or practical. Participants spent a mean of 11.2 hours (sd 6.2) attending live education (7.1 hours attending lectures and 4.1 hours attending practicals) and 10.5 hours (sd 9.3) going through the web materials. The amount of time spent on attending live education was negatively correlated with the amount of time spent on studying distance learning materials (Pearson correlation −0.5; p < 0.001).

During the course, all lectures and practicals were offered both live and as a distance learning version; i.e. participants were given the possibility to choose between live and distance education. Reasons to attend or not to attend the lectures and practicals are summarized in Table 1. The most important reasons to attend live education was that “it was easier to stay focused during lectures” (50%), and “to ask questions during practicals” (50%). The most important reason *not* to attend lectures or practicals was “a lack of time” (52%, and 61%, respectively). All participants indicated that they appreciated the possibility to choose between live and distance education. Out of the 54 participants, 26 (48%) indicated they appreciate the flexibility that comes with it (e.g., “it allows me to combine the course with clinical duties” and “it’s nice to manage your own time schedule”). Five participants explicitly indicated that they used the distance learning materials to review and prepare for the test. Four stated that it was convenient since they didn’t have to travel to attend the course. Reasons to come to the live education included that participants perceived that live education offers more possibilities to ask questions (5 participants) and that there was more/better explanation during live education (1 participant). One participant stated that “distance education cannot be considered as an adequate replacement of live education for such a difficult topic”.

**Table 1:** Reasons to attend live lectures and practicals among participants in an MSc epidemiology course on confounding and effect modification

***Reasons to attend live education***	**Lectures**	**Practical**
	**(n = 48)**	**(n = 38)**
General preference for live instead of distance education	21 (44%)	20 (53%)
Easier to stay focused during live education	24 (50%)	11 (29%)
Preference for live education to understand difficult topics	19 (40%)	18 (47%)
Easier to ask questions during live education	10 (21%)	19 (50%)
Afraid to miss essential information when not going to live education	19 (40%)	13 (34%)
Other reason	7 (15%)	7 (18%)
***Reasons not to attend live education***	**(n = 27)**	**(n = 31)**
No time	14 (52%)	19 (61%)
Already understanding of the topic, i.e., no perceived need to attend live education	2 (7%)	1 (3%)
General preference for distance instead of live education	12 (44%)	9 (29%)
Easier to stay focused during distance education	7 (26%)	4 (13%)
Preference for distance education to understand difficult topics	3 (11%)	2 (6%)
Other reason	4 (15%)	0 (0%)

Forty-six participants completed the questionnaire on learning styles, of which the results are presented in Table 2. The learning style of the majority of the participants was active (61%), sensing (61%), visual (83%), and sequential (54%). Learning styles were not associated with the amount of time spent on live or distance education.

**Table 2:** Learning styles of participants in an MSc epidemiology course on confounding and effect modification, and the amount of time spent on live and distance education

**Learning style**	**Number (%) of participants**	**Mean (sd) number of hours spent on live education**	**Mean (sd) number of hours spent on distance education**
	**(n = 46)**		
Active	28 (61%)	12.0 (6.2)	9.9 (8.8)
Reflective	18 (39%)	10.7 (5.8)	9.6 (9.3)
Sensing	28 (61%)	12.0 (6.1)	10.9 (10.4)
Intuitive	18 (39%)	10.6 (6.0)	8.2 (6.0)
Visual	38 (83%)	11.6 (5.6)	10.2 (8.5)
Verbal	8 (17%)	10.8 (8.0)	8.0 (10.9)
Sequential	25 (54%)	12.3 (5.8)	10.3 (9.6)
Global	21 (46%)	10.4 (6.3)	9.1 (8.1)

Abbreviations. *sd*: standard deviation.

## Discussion

Participants in the MSc Epidemiology course on confounding and effect modification highly appreciated the possibility to choose between live and distance education, because it offered them flexibility, for example to combine the course with clinical duties. Main reasons to attend live education were that it was considered easier to ask questions and to stay focused during live education.

We did not observe any relation between learning styles and participation in either live or distance education. This is in line with other studies in the medical field. For example, also in a study among 93 medical students following an ECG-course, the use of an optional web-based ECG learning program was not related to learning styles [5]. In a study among 236 osteopathic medical students, learning styles seemed to be related to the use of online learning materials: active and intuitive learners were significantly more likely to use online study materials compared to reflective and sensing learners, respectively. On the other hand, in the same study the use of online study materials was not materially different between visual and verbal learners, and between sequential and global learners [6].

We did not perform a study to assess the relation between live vs. distance learning and the effectiveness of learning. The main reason for this being that we would like to observe the behaviour of student if they are offered the choice between the two modes of education. Because the questionnaires were anonymous, we were unable to relate the scores on the final exam to e.g. participation in live and distance education. Observational studies suggest that e-learning may improve student learning [7,8]. This was however not confirmed by a randomized trial [9,10]. It is hard to draw general conclusions on the effectiveness of live vs. distance learning given the large number of possibilities to implement live and distance learning [2].

Even though this study was conducted within a single group of only 54 students, it provides valuable information on a group that is not routinely included in research on learning styles or distance learning. Postgraduate Epidemiology students often combine their education with other activities including clinical duties, for which reason the offered flexibility of the course (i.e., the choice offered between live and distance learning) was highly appreciated. The convenience of distance learning has been stressed before [10].

There are several limitations of our study that need to be addressed. Firstly, information was self-reported and collected through questionnaires. This may have resulted in measurement error of, for example, the amount of time participants studied the distance learning materials. Unfortunately, the amount of time participants spent on accessing the materials was not automatically recorded. Secondly, we conducted our study in a relative small population (54 participants) in a short course of only 5 days. It is therefore unclear to what extent our results can be generalized to courses with more participants, or courses with a longer duration.

Distance learning may play a role in epidemiology courses on different topics, for the reasons that apply to all kinds of education: it allows to teach more students at once, and it allows participants to study at a time and place that is the most convenient. The participants in the course described here, used the video lectures to review the course contents rather than as a substitute for live education. The distance learning materials that were offered during the course were mainly based on video lectures and video manuals for the practicals, which could be a reason why distance learning materials were not considered an appropriate substitute for live education. In addition, the contents of the course was often perceived as difficult by participants and they were under the impression that it was easier to ask questions during live sessions than during distance education (e.g., through the e-learning environment (website) or by email). This suggests that the implementation of distance learning in epidemiology courses requires accessible and user-friendly tools to ask questions and interact with instructors.

## Conclusion

Distance learning may play an important role in epidemiology courses, since it allows participants to study whenever and wherever they prefer, which provides the opportunity to combine courses with other (e.g. clinical) obligations. An important requirement for distance learning education appears to be the possibility to ask questions and to interact with instructors.

## Competing interests

The authors declare that they have no competing interests.

## Authors’ contributions

Both authors contributed to the conception of this manuscript. RG conducted the statistical analyses and drafted the manuscript. MK critically revised the manuscript for important intellectual content. Both authors read and approved the final manuscript.

## Pre-publication history

The pre-publication history for this paper can be accessed here:

http://www.biomedcentral.com/1472-6920/13/93/prepub
